# Immediate Background Color Choice in Male and Female *Poecilia reticulata*

**DOI:** 10.3390/ani16132019

**Published:** 2026-07-02

**Authors:** Haoran Liu, Miao Xiang, Sushuang Liu, Xinye Wu, Jikai Ding, Jia Wu, Ruijun Wang, Yaoyu Feng, Yang Li

**Affiliations:** 1 College of Life Sciences, Huzhou University, Huzhou 313000, China; lhr020620@dingtalk.com (H.L.); wxyray1108@163.com (X.W.); adjkemail@163.com (J.D.); 02753@zjhu.edu.cn (R.W.); 2 Yangtze River Fisheries Research Institute, Chinese Academy of Fishery Sciences, Wuhan 430223, China; xiangmiaoihb@163.com; 3 Freshwater Fisheries Research Center, Chinese Academy of Fishery Sciences, Wuxi 214081, China; 4 School of Life and Health Sciences, Huzhou College, Huzhou 313000, China; liu@zjhzu.edu.cn; 5 College of Forestry and Biotechnology, Zhejiang A&F University, Hangzhou 311300, China; 18647843537@163.com; 6 Anji County Forestry Bureau, Huzhou 313300, China; wujia123666@163.com

**Keywords:** guppy, color choice, immediate behavioral response, sex-independent

## Abstract

*Poecilia reticulata* is an important species in the global ornamental fish trade, known for its environmental adaptability and bright adult coloration. However, research on sex-dependent background color choice in this species remains limited, constraining the understanding of visual-environment requirements across sexes. In this study, we used a circular six-color maze (red, yellow, blue, green, black, and white) to independently test male and female *P. reticulata* at first sexual maturity. The results showed that both sexes spent proportionally more time in blue and green zones and less time in red zones, with no sex differences observed. This indicates a convergent pattern of immediate color choice between sexes at this stage. This cross-sex convergent choice pattern may reflect similar short-term visual-environment requirements between sexes. This study provides descriptive baseline data on immediate color choice, which can serve as a reference for subsequent investigations into long-term stable color preferences in this species. The sex-separated testing approach also offers a methodological reference for similar studies in other species.

## Simple Summary

*Poecilia reticulata* at first sexual maturity showed an immediate tendency to spend a higher proportion of time in blue and green zones and less time in red zones in a six-color choice maze, with no significant sex differences. This convergent pattern of color choice may reflect similar short-term visual-environment requirements between sexes, and can provide descriptive baseline data for future research on color preference.

## 1. Introduction

Background color serves as a core component of the visual environment and exerts important effects on fish physiology, behavioral patterns, and habitat selection [1,2,3,4]. Studies have shown that background color can influence fish through multiple pathways, including the modulation of stress levels [5], mitigation of aggressive behavior [6], and optimization of habitat use [7]. Different species exhibit distinct responses to background color: *Clarias magur* shows optimal growth in white environments, whereas *Pangasius pangasius* exhibits reduced survival under black backgrounds [8]; black backgrounds promote growth and survival in *Dawkinsia filamentosa* [9]; yellow-green light enhances feeding efficiency in juvenile *Tachysurus fulvidraco* [10]; and red backgrounds improve feed conversion in *Clarias macrocephalus* × *Clarias gariepinus* [11]. These interspecific differences indicate that background color is an important environmental factor affecting fish physiology and behavior. In aquaculture practice, background color can significantly influence production performance and individual behavioral expression [12,13]. When the color of rearing containers conflicts with a species’ innate preference, individuals often develop behavioral abnormalities [14], metabolic disorders, and chronic stress responses including immunosuppression [15], thereby compromising population health and production efficiency. For example, *Trachinotus blochii* exposed to non-preferred background colors shows elevated stress, reduced survival, and impaired growth [16], indicating that background color selection is critical for fish health and survival. However, existing studies have largely focused on endpoint indicators such as growth, survival, and stress levels under set background colors, while the research on fish background color choice remains relatively limited. Immediate behavioral choice data can provide a basis for understanding species-specific visual-environment requirements, and may serve as baselines for future studies on stable color preferences [3]. Notably, sex represents a key biological variable that may influence color choice behavior [17,18]. However, the current research has mainly focused on interspecific comparisons, and sex differences remain understudied. Males and females differ in physiology, endocrinology, and behavioral strategies [19,20], and these differences may lead to divergent visual-environment requirements. To date, whether background color choice patterns converge or diverge between sexes is poorly documented. Therefore, the descriptive documentation of sex-dependent immediate color choice can provide preliminary baseline data on convergent or divergent responses between sexes, serving as a reference for understanding long-term preference formation.

Color serves as an important visual cue in the behavioral regulation of fish. Numerous studies have demonstrated that fish can discriminate bait color and exhibit selective responses [21,22,23]. However, some studies have reported that, under specific environmental conditions, color differences may not significantly affect catch rates [24]. Meanwhile, behavioral and neurophysiological evidence indicates that most fish possess well-developed color vision systems [25]. Color choice is an important pathway for understanding fish behavioral responses. Immediate data obtained through short-term behavioral tests can provide a basis for describing species-specific behavioral distribution across different color environments, and can serve as a baseline for research on long-term stable preferences. These findings may also hold potential value for the future exploration of color-related applications. Regarding sex differences in visual signals, female Danio rerio rely on body coloration and chemical cues to identify males [26], and female Crenuchus spilurus show a strong preference for red ambient light [27]. Therefore, separately evaluating immediate color choice in males and females of sexually dimorphic species is important for understanding sex differences in visual-environment requirements.

The guppy (*Poecilia reticulata*) is a small ovoviviparous tropical freshwater fish native to northern South America and the Caribbean region [28]. This species accounts for approximately one quarter of the global freshwater ornamental fish market, owing to its small body size, high fecundity, and excellent environmental adaptability [29,30]. At approximately two months of age, guppies show clear sexual dimorphism and acquire reproductive capability [31,32]. As a widely cultured ornamental species with pronounced sexual dimorphism, research on its background color choice characteristics remains limited. In particular, whether males and females exhibit different background color choice behaviors is still poorly investigated. Based on this, the present study used two-month-old *P. reticulata* and employed a six-color choice maze to independently evaluate behavioral responses of male and female individuals toward six background colors. This study aimed to document immediate color choice behavior, providing baseline data for subsequent research on long-term stable background color preferences and establishing a methodological reference for sex-separated independent testing.

## 2. Materials and Methods

### 2.1. Fish Maintenance

*P. reticulata* of the mosaic strain were purchased from Guangdong Shuiyunjian Aquarium Co., Ltd. (Guangzhou, China). Fish were two months old. They were acclimated in a recirculating aquaculture system at Huzhou University. Upon acquisition, fish were sexed based on external morphology. Females display a rounded, fan-shaped anal fin and a fuller abdomen. Males have a rod- or needle-shaped anal fin and a slenderer body profile. They were separated into male and female groups for rearing [31,32]. Fish were fed a commercial diet twice daily (8:00 and 18:00) before the experiment. No significant sex difference in body length was observed (*p* > 0.05), with mean lengths of 17.2 ± 1.18 mm (males) and 17.1 ± 1.23 mm (females) (Table 1).

Well water was filtered, aerated in a reservoir for 48 h, and then used as culturing water in the indoor system. Conditions were maintained as follows: temperature 21.4 ± 0.3 °C, dissolved oxygen 7.48 ± 0.13 mg/L, photoperiod 12L:12D, and light intensity 0–500 lux [33]. Connected tank bottoms were cleaned by water replacement after each individual trial to eliminate residual odor interference [5,14]. Behavioral observations were made daily from 8:00 to 18:00. Since color depends on light reflection [34], nighttime conditions render backgrounds black and unidentifiable; thus, nocturnal tests were not performed, and artificial noise was carefully avoided.

### 2.2. Experimental Apparatus

An optimized circular six-color choice maze was used as the behavioral test apparatus. The circular tank was equally divided by six square partitions (20 cm length) into six fan-shaped zones of equal area, with an 18 cm diameter central area. The acclimation column was cylindrical (10 cm diameter × 20 cm height), and water depth was maintained at 18 cm. Color stimuli were produced by surface light reflection rather than wavelength-specific emission. Hue and brightness therefore covaried across the six zones, and behavioral responses were assessed against these combined visual properties. Waterproof stickers of the following six colors were applied to the respective zones: red (RGB: 255, 0, 0), green (RGB: 0, 255, 0), black (RGB: 0, 0, 0), yellow (RGB: 255, 255, 0), white (RGB: 255, 255, 255), and blue (RGB: 0, 0, 255).

**Figure 1:**
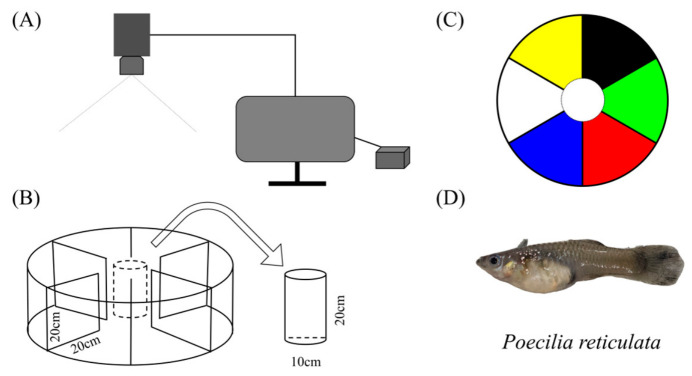
Animal behavior observation system and experiment fish: (**A**) infrared camera, (**B**) test tank with colors, (**C**) test tank (top view), and (**D**) *Poecilia reticulata*.

A circular heating mat matching the base area was placed at the bottom of the maze to maintain water temperature. A Sony LYT900 4K camera was positioned directly above the maze for real-time recording. The observation system also included an infrared night vision network camera (MJSXJ27CM) and a monitor (P27QCB-RG), with the camera fixed 2 m above the water surface (Figure 1) [35].

### 2.3. Experimental Methods

Fish were fasted for 24 h prior to the experiment. This was done to standardize satiation levels and ensure comparable activity motivation across individuals. It also minimized behavioral variability caused by differential feeding states. Previous studies have reported that a 24 h fasting period does not affect the survival rate of *P. reticulata* [36], indicating that this species tolerates such fasting duration well. Each fish was then placed into the cylindrical acclimation column. The column extended above the water surface. The experimenter stood outside the column and slowly removed the circular partition from the top after a 2 min acclimation period. The fish could not see the experimenter. This minimized disturbance from human presence. Before each trial, the angular position of the colored apparatus relative to the experimenter was randomly changed. This controlled for light direction, intensity, and position bias. The six background colors were assigned to fixed zones throughout the experiment. The testing sequence was also randomized. The fish was allowed to move freely and make choices within the apparatus. Behavior was continuously recorded by camera for 10 min. After that, the experiment ended and the video was saved. Each fish was used only once.

### 2.4. Data Analysis

Time to first exploration (s) is the time from the start of the experiment to the first entry of the fish’s geometric center into any color zone. It was used to evaluate the difference in the initiation speed of exploratory behavior between the two sexes.

For each individual in both sex groups, the duration and number of visits to each background color were recorded over 10 min. These values were calculated as follows:

P_t_ = t/T × 100

where P_t_ is the percentage of cumulative duration (%) of the experimental fish under different colors, t is the cumulative dwell time per fish under each color, and T is the total cumulative duration per fish across all six colors.

P_f_ = f/F × 100

where P_f_ is the percentage of counts (%) of experimental fish under each color, f is the visit frequency per fish under each color, and F is the total counts per fish across all six colors.

Time to first exploration was compared between male and female *P. reticulata*. This was used to assess the initial propensity to initiate exploration, irrespective of color zone. At the same time, residence time and visit frequency in each color zone were compared. This helped identify relative color choice patterns. Before analysis, normality was assessed using the Shapiro–Wilk test. None of these tests supported a normal distribution for residence time or visit count data. Therefore, non-parametric methods were used for all subsequent analyses. Descriptive statistics are reported as mean ± S.E. Within each sex, color choice was analyzed using the Kruskal–Wallis H test to compare duration percentage and count percentage across the six background colors. This assessed whether males (and, separately, females) showed differential residence or visit patterns among the six colors. When the Kruskal–Wallis test was significant, Dunn’s post hoc test with Bonferroni correction was used for pairwise comparisons. In addition, the Scheirer–Ray–Hare test was used to formally test the sex-by-color interaction on duration and count percentages. This determined whether the overall color choice ranking differed between sexes. Sex differences in time to first exploration were compared using the Mann–Whitney U test, to assess whether the two sexes differed in their propensity to initiate exploration. All statistical analyses were performed using GraphPad Prism 10.1 and SPSS 26.0. The significance level was set at *p* < 0.05.

## 3. Results

### 3.1. First Time Selection and Time to First Exploration

**Figure 2:**
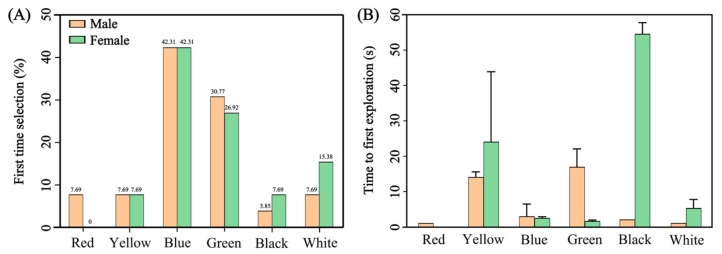
Percentage of first time selection (**A**) and time to first exploration (**B**) of *Poecilia reticulata* under six background colors.

Following acclimation column removal, the first-choice color proportions were analyzed for both male and female *P. reticulata*. Blue and green backgrounds were the most frequently selected first choices in both groups, whereas red was the least selected (Figure 2A). Time to first exploration was then compared. Within 10 s of baffle removal, 76.9% of fish became active and selected a background color. The maximum recorded latency was 51 s; yet, all individuals commenced exploration within 1 min. The mean times to first exploration were 7.73 ± 2.11 s and 8.31 ± 3.15 s for males and females, respectively, both under 10 s. The Mann–Whitney U test revealed no significant difference in exploration initiation speed between sexes (*p* > 0.05), indicating that males and females both exhibit rapid exploration behavior (Figure 2B).

### 3.2. Duration of Male and Female Poecilia reticulata

**Figure 3:**
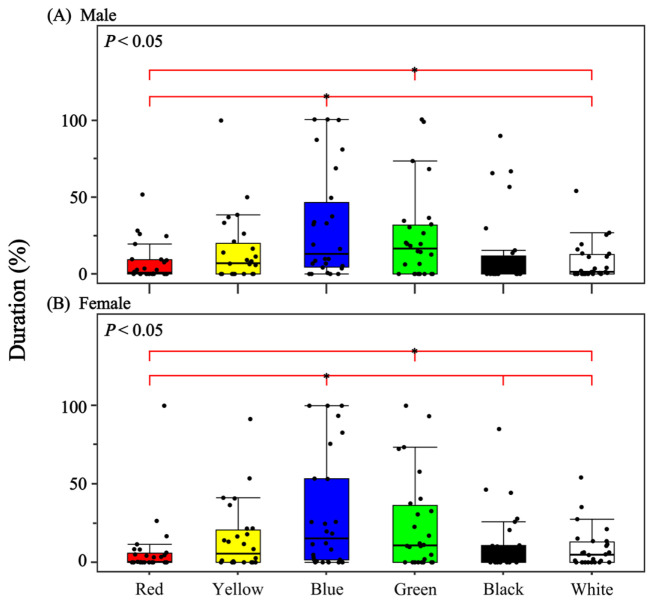
The duration (%) in different background colors of *Poecilia reticulata* different sex groups: (**A**) male group, and (**B**) female group. * indicate significant difference (*p* < 0.05).

In males, residence duration proportions varied significantly across color zones (Kruskal–Wallis, H = 12.37, *p* < 0.01). The order was blue (31.42 ± 7.82%) > green (24.58 ± 6.01%) > yellow (15.13 ± 4.32%) > black (12.19 ± 3.92%) > white (9.05 ± 2.56%) > red (7.64 ± 3.89%). Post hoc tests showed no significant difference between blue and green (*p* > 0.05), but both were significantly higher than red and white (*p* < 0.05); no other significant differences were found (*p* > 0.05) (Figure 3A, Table A1).

Females also showed significant differences in residence duration proportions across color zones (Kruskal–Wallis, H = 15.38, *p* < 0.01), with a trend similar to males. The order was blue (31.18 ± 6.96%) > green (24.25 ± 5.77%) > yellow (14.96 ± 4.38%) > black (14.25 ± 4.96%) > white (7.87 ± 2.45%) > red (7.50 ± 2.47%). Post hoc results were largely consistent with males, except that females spent significantly more time in blue than in black (*p* < 0.05), a difference not observed in males (Figure 3B, Table A1).

The Scheirer–Ray–Hare test showed no significant sex-by-color interaction (*p* > 0.05), indicating that males and females showed the same choice pattern for residence time across the six background colors. There was no sex-dependent difference in color choice.

### 3.3. Counts of Male and Female Poecilia reticulata

**Figure 4:**
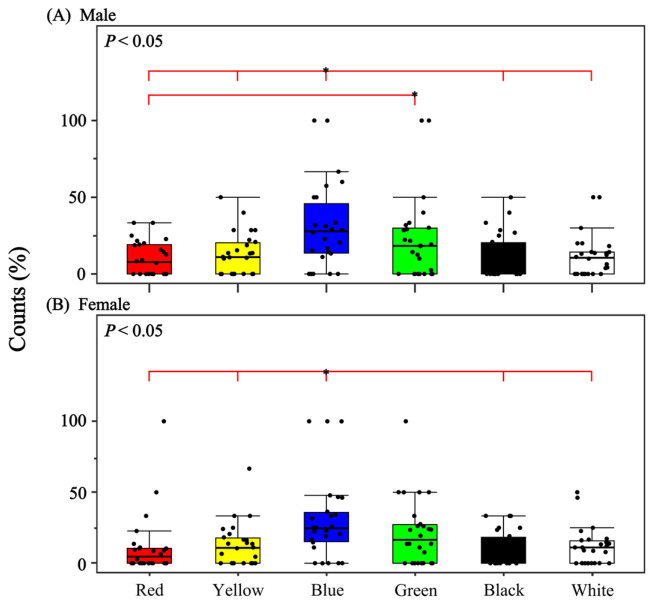
The counts (%) in different background colors of *Poecilia reticulata* different sex groups: (**A**) male group, and (**B**) female group. * indicate significant difference (*p* < 0.05).

In males, proportional visit counts varied significantly across color zones (Kruskal–Wallis, H = 18.51, *p* < 0.01). Blue (30.89 ± 5.72%) and green (21.68 ± 4.61%) zones received the highest visit counts, followed by yellow (12.64 ± 3.01%), white (11.89 ± 2.56%), red (11.49 ± 4.23%), and black (11.41 ± 2.31%). Post hoc tests showed no significant difference between blue and green (*p* > 0.05), but both were significantly higher than red (*p* < 0.05). Moreover, blue zone visit counts were significantly higher than those of yellow, black, and white zones (*p* < 0.05) (Figure 4A, Table A1).

Females also showed significant differences in proportional visit counts across color zones (Kruskal–Wallis, H = 15.87, *p* < 0.01). Blue (31.07 ± 5.48%) and green (22.39 ± 5.30%) zones had the highest counts, followed by yellow (12.92 ± 2.70%), black (11.84 ± 2.84%), white (11.73 ± 2.73%), and red (10.05 ± 2.20%). The overall trend mirrored that of males, except that, in females, the green zone visit counts were not significantly higher than those of the red zone (Figure 4B, Table A1).

The Scheirer–Ray–Hare test showed no significant sex-by-color interaction (*p* > 0.05), indicating that males and females showed the same choice pattern for visit counts across the six background colors. There was no sex-dependent difference in color choice.

## 4. Discussion

### 4.1. Rapid Exploration, Color Zone Selection, and Potential Mechanisms

It was found that 76.9% of the experimental fish entered the target color zone within 10 s of acclimation column removal, and all individuals completed their first exploration within 1 min, demonstrating active spatial exploration motivation. No significant difference was observed between the sexes in the initial response. This rapid entry behavior into color zones contrasts sharply with the cautious avoidance behavior exhibited by *D. rerio* when facing novel backgrounds [37], and may be attributable to the high heterogeneity of water body and substrate colors in the natural habitat of guppies [38,39,40], enabling the rapid localization of suitable shelters [41]. Furthermore, given that *P. reticulata* shows high exploration motivation under low-predation-risk conditions [42], this rapid exploratory behavior more likely reflects a spontaneous tendency in such contexts rather than a stable preference for specific colors. However, this is not the only interpretive perspective. It should be particularly noted that the first-choice color metric is highly susceptible to multiple confounding factors, including the fish’s orientation at partition removal, immediate escape routes, startle responses, and the individual’s initial body direction [43,44,45]. In this study, the high proportion (76.9%) of fish entering a color zone within 10 s of partition removal more likely stemmed from strong immediate exploratory motivation or stress-induced escape responses [46], rather than a stable preference for specific background colors. Therefore, although both sexes showed a higher proportion of first entry into blue–green zones and a lower proportion into red zones at the group level, these data may only reflect individuals’ immediate exploratory tendencies or stress responses in a novel environment, and cannot fully represent a stable preference for background colors.

Compared with first choice, residence time and visit frequency serve as cumulative behavioral indicators that can be used to describe behavioral distribution patterns of individuals across different color zones during the test period. Most studies also adopt such indicators to assess color choice characteristics [47]. At the group level in this study, both male and female individuals showed significantly higher residence proportions and visit frequencies in blue–green zones, and significantly lower values in red zones. This indicates a convergent pattern of immediate color zone choice between sexes. The formation of such choice patterns reflected by cumulative behavioral indicators may involve factors at multiple levels. On one hand, species-specific visual physiological characteristics, such as the high sensitivity of *P. reticulata* to the blue–green spectral range [48,49], combined with the fact that color itself is determined by the reflection of specific wavelength light from object surfaces [34], may influence behavioral responses toward particular colored light and corresponding backgrounds. On the other hand, the requirements for crypsis through matching body coloration with background environments [50,51], the demands for target recognition efficiency during foraging [33], and the degrees of habitat familiarity [47,52] may all lead to the selection or avoidance of certain colors, collectively contributing to the formation of this background color choice. These multi-level factors may have collectively influenced the immediate color choice patterns observed in this study. These immediate choice data can serve as baseline references for subsequent investigations into stable background color preferences, and may provide a starting point for subsequent research on the association between color environments and spatial distribution behavior.

### 4.2. Sex-Independent Background Color Choice and Individual Differences

A cross-sex quantitative analysis revealed that male and female *P. reticulata* exhibited consistent color choice, with blue residence proportions of 31.42 ± 7.82% and 31.18 ± 6.96%, and green proportions of 24.58 ± 6.01% and 24.25 ± 5.77%, respectively. This result contrasts with some previous reports: for example, male and female *D. rerio* show divergent background color choice behaviors [18], and sex differences in color sensitivity have also been reported in Lake Malawi cichlids [53]. By comparison, both sexes in this study showed similar immediate color zone choices, which may reflect comparable short-term visual-environment requirements between males and females at this stage. This convergent color selection may carry certain environmental adaptive significance. The selection for background colors similar to body coloration is consistent with the results from previous studies [47,54]—that is, the selection for colors similar to body coloration rather than colors producing a high contrast with body coloration, which fits the explanation of crypsis [55]. Furthermore, the visual system of *P. reticulata* shows a high sensitivity to the blue–green spectral range [48,49], and this species feeds mainly on organic detritus, small invertebrates, and benthic algae in the wild [56]. These food items typically reflect long-wavelength light and appear brown or brownish. Existing studies have indicated that this spectral sensitivity may be related to the foraging behavior of this species [57], and, under blue–green background conditions, the chromatic contrast formed between brown food items and the blue background is more pronounced, which may help enhance visual recognition efficiency during foraging [58,59,60], enabling individuals to rapidly locate and identify prey. Moreover, increasing evidence suggests that red, as a relatively uncommon background color in natural aquatic environments, is often associated with stress responses [61,62,63,64], which may explain why both sexes showed an avoidance of red zones. In summary, both male and female *P. reticulata* spent significantly more time in blue–green zones and markedly avoided red zones, and this convergent pattern of immediate color choice may be closely related to their visual physiological characteristics and foraging requirements.

It is worth noting that the individual differences in residence time proportion and visit count in specific color zones were substantial. Such differences may be related to the variation in personality traits such as boldness and exploration tendency among individuals [65,66]. Previous studies have also shown that individuals differing in boldness often exhibit large differences in background selection [67,68]. Bolder individuals tend to be more active and more willing to take risks in exploration, whereas shy individuals are relatively conservative [69]. In addition, differences in exploration tendency may also be an important factor affecting individual color choice, as different individuals may possess varying degrees of exploration tendency. In guppies, the exploration tendency and boldness may not be independent of each other [70,71]; that is, bold individuals tend to also be more exploratory, while shy individuals show the opposite pattern [72]. However, despite the potentially large behavioral differences among individuals, these did not obscure the overall longer cumulative residence time in blue–green zones and marked avoidance of red zones exhibited by male and female individuals at the group level.

In summary, this study reveals the immediate background color choice characteristics of *P. reticulata*. Both sexes showed higher residence proportions in blue–green zones and an avoidance of red zones, with a high similarity in this behavioral pattern between sexes. Immediate color choice data serve as a prerequisite basis for long-term preference assessment, and can provide baseline references for the subsequent exploration of stable color preference characteristics. Future research can further validate long-term preference characteristics on this basis, to explore ecological and behavioral implications of such preferences. Meanwhile, the sex-separated independent testing method adopted in this study can provide an experimental reference for similar studies in other sexually dimorphic fish species, and may be extended to more species to compare convergent and divergent patterns of color choice across species.

### 4.3. Limitations and Future Directions

In this study, we evaluated the immediate color choice of *P. reticulata* toward six background colors under short-term test conditions. The results showed similar color choice patterns between sexes. However, this study only captured the initial choice state without prior specific color conditioning. Previous studies have shown that mate color choice in adult *P. reticulata* can be significantly reversed within seven days following dietary color conditioning [73]. Whether this plasticity in mate color choice extends to background color choice, or even to long-term stable background color preferences, remains unclear. The findings of this study can provide baseline data for future investigations into long-term stable background color preferences and the plasticity of color choice and preference. Subsequent research can build on this to examine how initial background color choices change under prolonged exposure to specific color environments, and whether the length of the acclimation period affects the expression of long-term stable preferences. In addition, it is also of value to assess whether background color choice differs across developmental stages, as visual physiological characteristics and ecological demands may vary with age.

Nevertheless, this study has several limitations. First, the characterization of color stimuli is constrained. The six background colors were produced by surface light reflection from the sticker material. However, RGB coordinates are designed for human vision. *P. reticulata* possesses a more complex visual system. Its retina contains multiple opsin types with unique peak sensitivities [40]. In addition, we did not quantitatively assess the visual system of the experimental fish. Without such data, we cannot separate the effects of brightness and chroma on color selection behavior. Furthermore, the six colors were assigned to fixed zones throughout the experiment; although potential directional bias was controlled by randomly rotating the angular orientation of the apparatus before each trial, this does not fully exclude the possibility of an inherent positional preference for particular zones. Future studies should consider this species’ opsin-based spectral sensitivity to clarify the relationship between brightness and chroma, and randomly reassign colors to different zones in each trial to further control for positional effects. Second, the limited sample size (n = 52; 26 per sex) limits the precision for detecting subtle parameter-level differences. We encourage future studies to employ larger sample sizes for replication. This would facilitate the adoption of Friedman tests or linear mixed-effects models to more rigorously account for the repeated-measures structure.

## 5. Conclusions

In this study, two-month-old *P. reticulata* were used to independently evaluate background color choice behavior in male and female individuals using a six-color choice maze. Color choice patterns reflected by cumulative indicators showed that both male and female individuals exhibited higher residence times and visit frequencies in blue–green zones, and markedly avoided red zones, with a similarity in this behavioral pattern between sexes. This convergent color choice pattern may be related to factors such as visual physiological characteristics, body coloration crypsis requirements, and foraging recognition efficiency. Based on these immediate choice characteristics, this study can provide baseline data for the future exploration of long-term stable background color preferences, and may offer potential directions for further research on color-guided behaviors. In addition, the sex-separated testing method adopted in this study can provide an experimental reference for evaluating sex differences in color choice in other fish species, and may be extended in the future to species with divergent sex-specific preferences to conduct comparative studies on sex-specific visual behavior.

## Figures and Tables

**Table 1:** Total length (±S.E.) and Weight (±S.E.) of *Poecilia reticulata* in the present study during the background color experiments.

Species	Sex	Number	Total Length/mm	Weight/g
*Poecilia reticulata*	Male	26	17.2 ± 1.18	0.48 ± 0.05
	Female	26	17.1 ± 1.23	0.47 ± 0.06

## Data Availability

Dataset available on request from the authors.

## Appendix A

**Table A1:** Mean Percentage (±S.E.) for visit frequency and duration of *Poecilia reticulata* across six background colors under male and female groups.

Conditions		Background Colors					
		Red	Yellow	Blue	Green	Black	White
Male	Counts	11.49 ± 4.23%	12.64 ± 3.01%	30.89 ± 5.72%	21.68 ± 4.61%	11.41 ± 2.31%	11.89 ± 2.56%
	Duration	7.64 ± 3.89%	15.13 ± 4.32%	31.42 ± 7.82%	24.58 ± 6.01%	12.19 ± 3.92%	9.05 ± 2.56%
Female	Counts	10.05 ± 2.20%	12.92 ± 2.70%	31.07 ± 5.48%	22.39 ± 5.30%	11.84 ± 2.73%	11.73 ± 2.73%
	Duration	7.50 ± 2.47%	14.96 ± 4.38%	31.18 ± 6.96%	24.25 ± 5.77%	14.25 ± 4.96%	7.87 ± 2.45%
